# Haemolysis in G6PD Heterozygous Females Treated with Primaquine for *Plasmodium vivax* Malaria: A Nested Cohort in a Trial of Radical Curative Regimens

**DOI:** 10.1371/journal.pmed.1002224

**Published:** 2017-02-07

**Authors:** Cindy S. Chu, Germana Bancone, Kerryn A. Moore, Htun Htun Win, Niramon Thitipanawan, Christina Po, Nongnud Chowwiwat, Rattanaporn Raksapraidee, Pornpimon Wilairisak, Aung Pyae Phyo, Lily Keereecharoen, Stéphane Proux, Prakaykaew Charunwatthana, François Nosten, Nicholas J. White

**Affiliations:** 1 Shoklo Malaria Research Unit, Mahidol–Oxford Tropical Medicine Research Unit, Faculty of Tropical Medicine, Mahidol University, Mae Sot, Thailand; 2 Centre for Tropical Medicine and Global Health, Nuffield Department of Medicine, University of Oxford, Oxford, United Kingdom; 3 Macfarlane Burnet Institute for Medical Research and Public Health, Melbourne, Victoria, Australia; 4 Centre for Epidemiology and Biostatistics, Melbourne School of Population and Global Health, The University of Melbourne, Melbourne, Victoria, Australia; 5 Mahidol–Oxford Tropical Medicine Research Unit, Faculty of Tropical Medicine, Mahidol University, Bangkok, Thailand; 6 Department of Clinical Tropical Medicine, Faculty of Tropical Medicine, Mahidol University, Bangkok, Thailand; Liverpool School of Tropical Medicine, UNITED KINGDOM

## Abstract

**Background:**

Radical cure of *Plasmodium vivax* malaria with 8-aminoquinolines (primaquine or tafenoquine) is complicated by haemolysis in individuals with glucose-6-phosphate dehydrogenase (G6PD) deficiency. G6PD heterozygous females, because of individual variation in the pattern of X-chromosome inactivation (Lyonisation) in erythroid cells, may have low G6PD activity in the majority of their erythrocytes, yet are usually reported as G6PD “normal” by current phenotypic screening tests. Their haemolytic risk when treated with 8-aminoquinolines has not been well characterized.

**Methods and Findings:**

In a cohort study nested within a randomised clinical trial that compared different treatment regimens for *P*. *vivax* malaria, patients with a normal standard NADPH fluorescent spot test result (≳30%–40% of normal G6PD activity) were randomised to receive 3 d of chloroquine or dihydroartemisinin-piperaquine in combination with primaquine, either the standard high dose of 0.5 mg base/kg/day for 14 d or a higher dose of 1 mg base/kg/d for 7 d. Patterns of haemolysis were compared between G6PD wild-type and G6PD heterozygous female participants. Between 21 February 2012 and 04 July 2014, 241 female participants were enrolled, of whom 34 were heterozygous for the G6PD Mahidol variant. Haemolysis was substantially greater and a larger proportion of participants reached the threshold of clinically significant haemolysis (fractional haematocrit reduction >25%) in G6PD heterozygotes taking the higher (7 d) primaquine dose (9/17 [53%]) compared with G6PD heterozygotes taking the standard high (14 d) dose (2/16 [13%]; *p* = 0.022). In heterozygotes, the mean fractional haematocrit reductions were correspondingly greater with the higher primaquine dose (7-d regimen): −20.4% (95% CI −26.0% to −14.8%) (nadir on day 5) compared with the standard high (14 d) dose: −13.1% (95% CI −17.6% to −8.6%) (nadir day 6). Two heterozygotes taking the higher (7 d) primaquine dose required blood transfusion. In wild-type participants, mean haematocrit reductions were clinically insignificant and similar with both doses: −5.8 (95% CI −7.2% to −4.4%) (nadir day 3) compared with −5.5% (95% CI −7.4% to −3.7%) (nadir day 4), respectively. Limitations to this nested cohort study are that the primary objective of the trial was designed to measure efficacy and not haemolysis in relation to G6PD genotype and that the heterozygote groups were small.

**Conclusion:**

Higher daily doses of primaquine have the potential to cause clinically significant haemolysis in G6PD heterozygous females who are reported as phenotypically normal with current point of care tests.

**Trial Registration:**

ClinicalTrials.gov NCT01640574.

## Abstract 2

In a cohort study within a clinical trial of radical cure regimens for malaria, Cindy S. Chu and colleagues assess the haemolytic risk associated with primaquine treatment among women heterozygous for glucose-6-phosphate dehydrogenase deficiency.

## Author Summary

**Why Was This Study Done?:**

- Primaquine is the only widely available treatment to eliminate latent liver stages of *P*. *vivax* malaria and thereby prevent relapse (radical cure).
- Primaquine can cause potentially severe haemolysis in glucose-6-phosphate dehydrogenase (G6PD)-deficient patients. Hemizygous males and homozygous females are identified reliably as G6PD deficient by current phenotypic testing; however, heterozygous females may have a normal or deficient result on G6PD rapid diagnostic phenotypic tests. G6PD heterozygous females who have a normal G6PD phenotypic test are still susceptible to clinically important haemolysis caused by primaquine.
- This study was designed to describe and quantify the haematocrit changes in G6PD Mahidol heterozygous females with a normal G6PD phenotype when taking primaquine for the radical cure of *P*. *vivax* malaria.

**What Did the Researchers Do and Find?:**

- The researchers administered a standard high-dose primaquine regimen (0.5 mg base/kg/d for 14 d) or an alternative higher-dose primaquine regimen (1 mg base/kg/d for 7 d) to participants infected with *P*. *vivax* malaria who tested as G6PD normal by the G6PD fluorescent spot test. Haematocrit levels were followed for 2 wk and compared with respect to the G6PD genotype, quantitative G6PD enzymatic activity, and initial parasitaemia.
- Higher daily doses of primaquine (1 mg base/kg/d) were associated with significant haemolysis in female participants who were heterozygous for Mahidol variant G6PD deficiency.
- The standard high dose primaquine (0.5 mg base/kg/d for 14 d) was well tolerated in all female participants who had a normal G6PD phenotypic test.

**What Do These Findings Mean?:**

- In the absence of close monitoring, daily doses higher than standard high dose primaquine (greater than 0.5 mg base/kg/d) should not be used in females who are heterozygous for a G6PD mutation that is similar to or more severe than the Mahidol variant.
- More information is needed on the haemolytic risk in G6PD heterozygous women and girls with different G6PD enzymatic activity levels.
- New rapid diagnostic tests for G6PD deficiency should be quantitative and include assessment of haemoglobin levels.
- Safer regimens of primaquine are needed for G6PD-deficient patients.

## Introduction

Primaquine is the only widely available drug that is effective against *P*. *vivax* hypnozoites, the latent forms that emerge from the liver to produce relapses of *P*. *vivax* malaria. The recommended regimen to prevent relapse of *P*. *vivax* malaria (called radical treatment) is primaquine given for 14 d at a daily dose of 0.25 to 0.5 mg base/kg. However, although widely recommended for radical treatment of *P*. *vivax* and *P*. *ovale* infections, primaquine is often not given. This is because primaquine causes haemolysis in glucose-6-phosphate dehydrogenase (G6PD)-deficient individuals [1]. While G6PD deficiency is very common in malaria-endemic areas, G6PD testing is generally unavailable because the standard point-of-care test requires appropriate reagents, electricity, trained staff, and quality controls.

The point-of-care diagnosis of G6PD deficiency is usually made by a phenotypic screening test in which the G6PD-mediated reduction of NADP+ to NADPH is measured semiquantitatively. The standard test is the fluorescent spot test (FST), which assesses the fluorescence of NADPH in a blood spot under ultraviolet light [2]. This identifies blood samples with ≳30%–40% activity as abnormal. It is considered safe to give primaquine (and other oxidant drugs) to persons who screen as having “normal” G6PD activity determined by the FST or other comparable rapid tests. The G6PD gene is on the X-chromosome. Hemizygous males and homozygous females have markedly reduced G6PD enzymatic activity and will therefore be identified reliably by these tests. However, in G6PD heterozygous females, random X-chromosome inactivation (Lyonisation) [3] results in red cell mosaicism. Individual red blood cells therefore express either a normal or deficient phenotype, so the overall blood G6PD activity level is an average determined by the relative proportions of the two red cell populations. The average proportion of deficient red cells in the population of heterozygotes is approximately half, but because random X-inactivation occurs early in embryogenesis, some women may have normal G6PD activity in the majority of their red cells, whereas others may have a majority of cells that are deficient. As a consequence, heterozygous females with a “normal” G6PD phenotype by the FST may have up to 60%–70% of their red blood cells that are G6PD deficient and susceptible to haemolysis by oxidant stresses.

Along the Myanmar border with northwestern Thailand, the prevalence of G6PD deficiency in males is 9%–18%. The Mahidol variant is most common (88% of all variants); other variants include Chinese-4, Viangchan, and Mediterranean type [4]. As part of a dose optimisation trial to assess potentially simpler primaquine regimens for the radical treatment of *P*. *vivax* malaria in patients screened as “G6PD normal,” we report patterns of haemolysis in relation to the daily dose of primaquine and the G6PD genotype.

### Ethics Statement

The trial was approved by the Faculty of Tropical Medicine, Mahidol University Ethics Committee (MUTM 2011–043, TMEC 11–008), and the Oxford Tropical Research Ethics Committee (OXTREC 17–11), and it was registered on the ClinicalTrials.gov website (NCT01640574).

## Methods

### Setting and Population

This nested cohort study was performed within a randomized nonblinded clinical trial in patients with acute *P*. *vivax* malaria. It was conducted by the Shoklo Malaria Research Unit (SMRU), which operates clinics extending over 120 km along the Thailand–Myanmar border. The clinics serve migrants and displaced persons of Burman and Karen ethnicities originating from as far as 30 km inside Myanmar.

### Participants and Trial Procedures

Patients more than 6 mo old with symptomatic *P*. *vivax* mono-infection confirmed by microscopy were diagnosed in outpatient clinics and enrolled in the study if they or the carers of children < 18 y old gave fully informed written consent. Patients were excluded if they were G6PD deficient by FST or were pregnant or breastfeeding an infant ≤ 6 mo old, had severe malaria, an allergy to trial drugs, haematocrit ≤ 25%, or received a blood transfusion within 3 mo, or could not comply with the trial requirements. Enrolment investigations included malaria smear, haematocrit, G6PD FST, and full blood count.

Randomisation to each of four treatment arms was computer generated in blocks of 20. Assignment of the participant to a treatment arm was centralized; a phone call from the clinic-based study staff was made to an independent staff member not involved with the trial. Trial codes were given sequentially, and written confirmation was provided.

Participants were randomized to one of the following treatment regimens:

1. Chloroquine (Medopharm, India) 25 mg base/kg divided over 3 d (CQ) and primaquine (Utopian, Thailand) 1 mg base/kg/d for 7 d (PMQ-1)
2. CQ and primaquine 0.5 mg base/kg/d for 14 d (PMQ-0.5)
3. Dihydroartemisinin-piperaquine (Sigma-tau, Italy); dihydroartemisinin 7 mg/kg and piperaquine 55 mg/kg, divided over 3 d (DP) and PMQ-1
4. DP and PMQ-0.5

Treatment with all drugs began on the day of enrolment. Doses were calculated using charts with predefined weight bands (S1 Appendix). All primaquine doses were given after food and were supervised. Participants were seen weekly after treatment was completed.

### Laboratory Procedures

Investigations included malaria smear, haematocrit, full blood count, G6PD phenotypic tests (described below), and G6PD genotyping for the prevalent Mahidol variant (487G>A). Capillary haematocrit was measured daily until day 7 and again on day 14. Haematocrit was measured on 30 μL of blood obtained via fingerstick using a heparinized capillary tube centrifuged at 10,000 rotations per minute (rpm) for 3 min and then read manually with a Hawksley Micro-Haematocrit reader. Anaemia requiring haematinic treatment was defined as an absolute haematocrit <30%. Haemoglobin variants were analysed using Capillary Electrophoresis (Capillarys2, SEBIA, France) in some patients with anaemia. The G6PD FST (R&D Diagnostic, Greece) was performed using 5 μL of blood mixed with 100 μL of reagents (containing haemolysing agents and NADP+ /G6P substrates). After 10 min of incubation at room temperature, a 15 μL aliquot was spotted on filter paper and allowed to dry in air. The spots were then examined under long-wavelength UV light (ca. 340 nm) to visualize the naturally fluorescent NAPDH; spots that fluoresced were classified as normal, and those that did not were classified as deficient. G6PD genotyping for Mahidol variant was performed on all female participants using an established PCR–RFLP protocol [5]. In order to explain differences in haemoglobin reductions observed in heterozygous female participants with the same genotype, quantitative assessment of G6PD activity was performed at steady state 6 to 8 wk later and repeated twice per patient on two additional follow-up samples. Spectrophotometric quantitative assessment of G6PD enzymatic activity [6] was performed using the Trinity kit assay (Trinity Biotech, Ireland) in duplicate 10 μL whole blood samples. The absorbance at 340 nm at 30°C was measured over 10 min by a UV spectrophotometer (UV-1800, Shimadzu, Japan) with an electronically controlled temperature compartment. Estimated G6PD activity was calculated as the arithmetic mean of three repeated assessments and expressed per gram of haemoglobin (IU/gHb), as is commonly reported, and also per red blood cell (IU/RBC), which is considered more accurate in anaemic patients [7]. Population median values and ranges for both assessments have been established previously in this population [8].

### Outcomes

The primary outcome of this analysis was the fractional haematocrit reduction up to day 14 after enrolment. Secondary outcomes included a description of the temporal patterns of haemolysis and characterisation of the effects of the initial parasitaemia and G6PD activity on haematocrit reduction. A symptom sheet including common complaints was reviewed daily with each participant during study drug administration. Adverse events occurring after treatment completion were then collected passively and reported until day 42.

### Bias

Laboratory data were processed by laboratory staff blinded to the clinical status of the participants. Subjective data (i.e., symptoms and adverse events) were collected by clinical study staff not involved with the study analysis. Because the main trial was not blinded, there may have been potential bias in extracting complaints of common adverse effects caused by primaquine; however, the G6PD genotype of all participants was unknown at the time of drug treatment.

### Sample Size

In this population, the prevalence of G6PD deficiency in males has been determined previously to be around 14% (varying from 9% to 18% depending on the area and/or ethnic group) [4]. Using this proportion as the allelic frequency and based on the Hardy-Weinberg principle, we expected 24% of females to be G6PD heterozygotes (varying from 15% to 30%) in the main trial. Because of the small sample size amongst heterozygote participants, we pooled the DP and CQ treatment regimens. Therefore, we analysed female participants in four groups:—by G6PD status—heterozygote and wild type, and—by primaquine dose—PMQ-0.5 and PMQ-1.

### Statistical Analysis

The hypothesis for this study was proposed before data collection, and the analysis was planned after data collection began. First, descriptive statistics were used to compare baseline participant characteristics between the four groups (as defined above). Then, a complete case analysis was performed; missing data were excluded. Haematocrit changes over time were normalized:

$$\mathrm{Fractional haematocrit change}=(hct_{\mathrm{day x}}-hct_{\mathrm{day 0}})/hct_{\mathrm{day 0}}$$

Unadjusted results were compared between groups and expressed as mean changes. A conservative estimate of a significant fractional haematocrit change was considered a priori to be +/− 15%—to take into account a technical variation of 10% and an actual 5% change [9]. However, fractional haematocrit changes of +/− 25% have been more commonly considered as clinically significant [10], so both were assessed. Multivariable linear regression adjusted for initial parasitaemia was used to assess the following:

1. differences between groups in the mean of the maximum fractional haematocrit reduction (without and then with a test for interaction between G6PD genotype and primaquine dose; the interaction was added to the analysis in response to peer review);
2. the differences between groups in the estimated change in the mean of the maximum fractional haematocrit reduction per 10-fold increase in parasitaemia (including tests for interaction between initial parasitaemia and G6PD genotype and between initial parasitaemia and primaquine dose);
3. the association between the maximum fractional haematocrit reduction and quantitative G6PD activity determined by spectrophotometry (including tests for interaction between G6PD activity and G6PD genotype and between G6PD activity and primaquine dose).

Pearson’s correlation was used to determine the relationship between the mean of the maximum fractional haematocrit reduction and G6PD activity for the heterozygote groups. Multivariable logistic regression was used to assess differences between groups in the proportions of participants with fractional haematocrit reductions >15% and >25% unadjusted for initial parasitaemia (including a test for interaction between G6PD genotype and primaquine dose) and the presence of absolute haematocrit reductions below 30% (the threshold for anaemia treatment). The likelihood ratio test was used to determine whether the interactions were significant. Univariable logistic regression was used to assess symptoms during primaquine administration and adverse events between groups. Of the participants analysed, none were lost to follow up by day 14. Statistical analyses were performed using Stata version 13 (StataCorp, College Station, Texas, United States).

## Results

**Fig 1:**
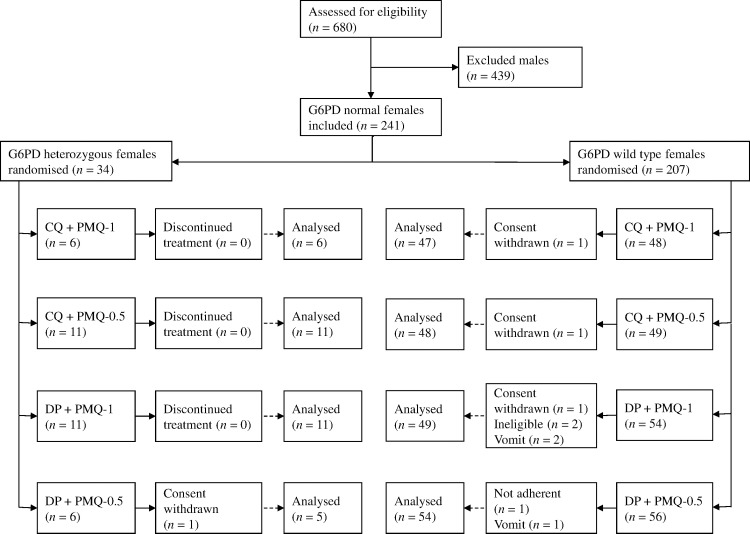
Trial diagram. CQ, chloroquine 25 mg base/kg divided over 3 d; DP, dihydroartemisinin 7 mg/kg and piperaquine 55 mg/kg divided over 3 d; PMQ-1, primaquine 1 mg base/kg/d x 7 d; PMQ-0.5, primaquine 0.5 mg base/kg/d x 14 d.

Between March 2012 and July 2014, 680 patients with acute *P*. *vivax* malaria and a normal G6PD FST were enrolled in the main trial. Details of all participants and the therapeutic outcomes will be reported elsewhere. Of the 241 female participants recruited, 34 (14%) were subsequently found to be G6PD Mahidol heterozygotes. One withdrew from the trial, leaving 33 with evaluable data (Fig 1). Symptoms, vital signs, and laboratory indices were similar in all treatment groups. G6PD heterozygous female participants taking PMQ-0.5 were younger (median 12 y [interquartile range (IQR) 7 to 27.5; range 1.5 to 45]) than heterozygous female participants taking PMQ-1 (19 y [IQR 14 to 27; range 6 to 38]) or wild-type female participants taking PMQ-1 (17 y [IQR 10.5 to 35; range 2 to 63]) or PMQ-0.5 (21 years [IQR 11 to 38; range 3 to 60]) (Table 1). Children less than 5 y old were under-represented in the heterozygous female PMQ-1 group.

**Table 1:** Participant characteristics.

Characteristic	Wild Type (*n* = 198)		Heterozygote (*n* = 33)	
	PMQ-1	PMQ-0.5	PMQ-1	PMQ-0.5
Total participants^a^, *n*	96	102	17	16
>15 y, *n* (%)	57 (60%)	60 (59%)	12 (71%)	6 (38%)
6 to 15 y, *n* (%)	31 (32%)	32 (32%)	5 (29%)	7 (44%)
≤5 y, *n* (%)	8 (8%)	10 (10%)	0	3 (19%)
Age (years), median (IQR, range)	17 (10.5–35, 2–63)	21 (11–38, 1.6–60)	19 (14–27, 6–38)	12 (7–27.5, 1.5–45)
History of fever, *n* (%)	94 (98%)	101 (99%)	17 (100%)	16 (100%)
Headache, *n* (%)	88 (92%)	95 (93%)	17 (100%)	12 (75%)
Dizziness, *n* (%)	59 (63%)	66 (65%)	13 (77%)	10 (63%)
Fatigue^b^, *n* (%)	17 (17%)	32 (32%)	7 (41%)	1 (7%)
Abdominal pain, *n* (%)	20 (21%)	27 (27%)	5 (29%)	3 (19%)
Anorexia, *n* (%)	35 (37%)	41 (40%)	9 (53%)	8 (50%)
Nausea, *n* (%)	25 (26%)	34 (33%)	9 (53%)	5 (31%)
Vomiting, *n* (%)	22 (23%)	28 (28%)	4 (24%)	1 (6%)
Palpitations, *n* (%)	29 (31%)	34 (34%)	6 (35%)	2 (13%)
Muscle pain, *n* (%)	49 (52%)	53 (53%)	10 (59%)	7 (44%)
Temperature ≥37.5°C, *n* (%)	47 (49%)	44 (43%)	8 (47%)	8 (50%)
Temperature (°C), median (IQR, range)	37.4 (36.9–38.3, 36–40.4)	37.3 (36.9–38.2, 36–40.1)	37.3 (37.1–38.4, 36.2–40.4)	37.5 (36.6–38.4, 36.1–40.5)
Heart rate (bpm), median (IQR, range)	90 (82–104, 64–160)	88 (80–100, 64–130)	86 (80–98, 68–108)	91 (85–106, 68–128)
Respiratory rate/min, median (IQR, range)	24 (22–26, 18–48)	24 (22–26, 20–40)	24 (22–24, 20–28)	23 (21–28, 20–36)
Systolic BP^c^ (mmHg), median (IQR, range)	100 (90–110, 80–190)	100 (90–110, 80–140)	100 (100–110, 90–120)	100 (90–120, 70–120)
Diastolic BP^c^ (mmhg), median (IQR, range)	60 (60–70, 50–100)	60 (60–70, 40–100)	70 (60–70, 50–80)	60 (60–80, 50–80)
Oxygen saturation (%), median (IQR, range)	99 (98–99, 96–100)	99 (98–99, 94–100)	99 (98–99, 97–100)	99 (98–100, 95–100)
Transcutaneous methaemoglobin^d^ (%), median (IQR, range)	0.6 (0.4–0.9, 0–9.0)	0.65 (0.3–1.1, 0–3.0)	0.5 (0.2–0.9, 0–1.2)	0.55 (0–1.0, 0–1.7)
Parasitaemia per μL, geometric mean (95% CI)	3,200 (2,397–4,271)	3,985 (2,973–5,343)	4,046 (1,939–8,445)	5,353 (2,785–10,290)
Gametocytaemia, *n* (%)	74 (80%)	82 (82%)	14 (82%)	12 (75%)
Enrolment haematocrit (%), mean (SD)	36.5 (4.1)	37.0 (4.3)	36.8 (4.3)	36.4 (4.8)
Day 14 haematocrit (%), mean (SD)	35.5 (3.2)^e^	35.7 (3.4)^f^	35 (3.3)^f^	34.3 (3.4)

BP, blood pressure; IQR, interquartile range; PMQ-1, primaquine 1 mg base/kg/d x 7 d; PMQ-0.5, primaquine 0.5 mg base/kg/d x 14 d; SD, standard deviation.

^a^Withdrawn participants were analysed if daily haematocrit data were available until day 7.

^b^G6PD heterozygous females in the PMQ-0.5 group were significantly less likely to complain of fatigue at enrolment (*p* = 0.028).

^c^Blood pressure was measured only in participants > 12 y old.

^d^Methaemoglobin measurements were not available in 16 participants.

^e^Number of missing data = 9.

^f^Number of missing data = 4.

### Haemolysis

The effect of primaquine dose on haemolysis was different in G6PD wild-type and G6PD heterozygous female participants (*p* = 0.0104 for interaction). There was no evidence of primaquine dose-dependent haemolysis in G6PD wild-type female participants when comparing PMQ-1 and PMQ-0.5, and there were no differences when comparing the CQ and DP groups (S1 Table). In contrast, primaquine was associated with clear dose-dependent haemolysis in G6PD heterozygous female participants. In the pooled analysis (CQ+DP) of G6PD heterozygous female participants, unadjusted for parasitaemia, 14/17 (82%) in the PMQ-1 group had fractional haematocrit reductions >15% compared with 6/16 (38%) G6PD heterozygous female participants in the PMQ-0.5 group (odds ratio [OR] 7.8 [95% CI 1.6 to 38.8]; *p* = 0.012). When comparing the proportions of G6PD heterozygous female participants with a fractional haematocrit reduction of >25%, the results were similar: 9/17 (53%) in the PMQ-1 group had fractional haematocrit reductions >25% compared with 2/16 (13%) G6PD heterozygous female participants in the PMQ-0.5 group (OR 7.9 [95% CI 1.4 to 45.8]; *p* = 0.022).

**Fig 2:**
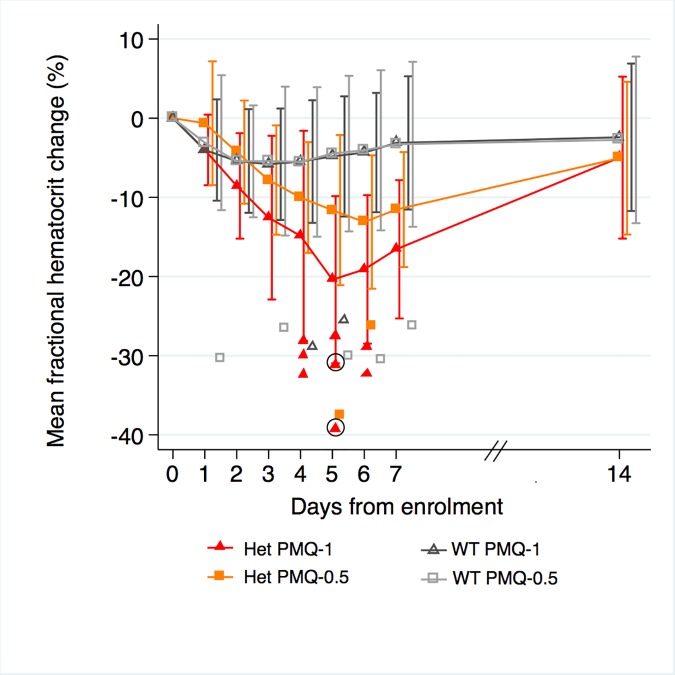
Mean fractional haematocrit changes over time in G6PD heterozygous and wild-type females taking PMQ-1 or PMQ-0.5. Line graph represents fractional haematocrit plotted as the mean (95% CI). Plotted shapes represent individuals with maximum fractional haematocrit reductions below −25%. Circled shapes represent individuals who received a blood transfusion.

**Fig 3:**
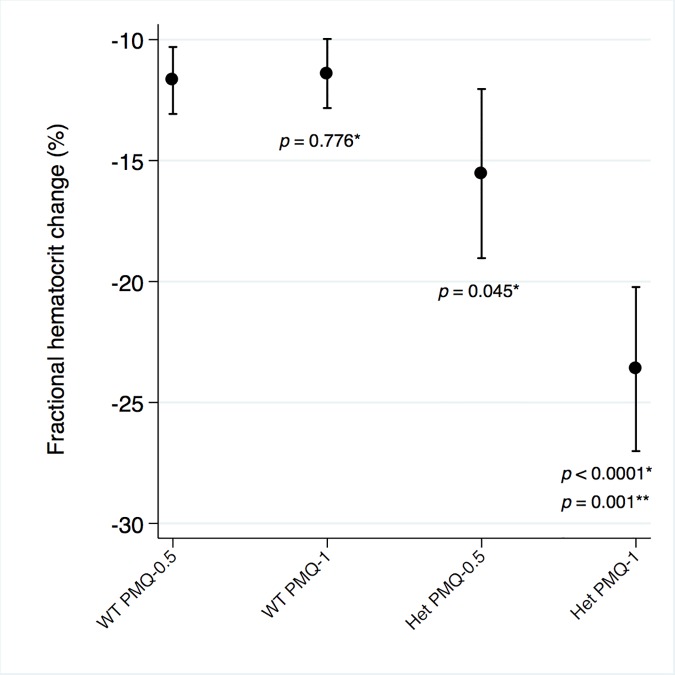
Mean maximum individual fractional haematocrit reductions in G6PD heterozygous and wild-type females taking PMQ-1 or PMQ-0.5. Het, heterozygote; WT, wild type. Maximum individual fractional haematocrit plotted as mean (95% CI) not adjusted for initial parasitaemia. *The comparator group is the wild-type PMQ-0.5 group. **Comparison between heterozygote PMQ-1 and heterozygote PMQ-0.5 group.

Among G6PD heterozygous female participants who took PMQ-1 with DP, the haematocrit reached its nadir on day 5 with a mean fractional reduction of −23.2% (SD 11.0) compared to −16.5% (SD 7.4) on day 6 in those taking PMQ-0.5. In the CQ group, the haematocrit reached its nadir on day 4 with a mean fractional reduction of −16.4% (SD 7.9) with PMQ-1 compared to −11.9% on day 5 (SD 11.1) with PMQ-0.5 (S1 Table). Irrespective of primaquine dose, by day 7 the mean fractional haematocrit rose again in all groups (except for three G6PD wild-type participants) despite continued primaquine administration (Fig 2 and S2 Table). In the pooled analysis, unadjusted for parasitaemia and including a test for interaction between G6PD genotype and primaquine dose, the effect of primaquine dose on haemolysis remained different in G6PD wild-type and G6PD heterozygous female participants (*p* = 0.0018 for interaction). The mean maximum individual fractional haematocrit reduction (mean of the individual nadirs) was similar between G6PD wild-type female participants taking PMQ-1 and PMQ-0.5, but in G6PD heterozygous female participants, it was significantly greater in the PMQ-1 group (−23.6% [95% CI −27.0% to −20.2%]) compared with the PMQ-0.5 group (−15.5% [95% CI −19.0% to −12.0%]), with a mean difference of −8.1% (95% CI −13.0% to −3.2%; *p* = 0.001) (Fig 3 and Table 2). When the same analysis was performed adjusting for parasitaemia, the effect of genotype and dose was reduced significantly, and the interaction of primaquine dose on haemolysis remained different in G6PD wild-type and G6PD heterozygous female participants (*p* = 0.0014 for interaction) (Tables 2 and 3). The estimated change in the mean maximum fractional haematocrit per 10-fold increase in initial parasitaemia in G6PD wild-type female participants (without testing for interaction between G6PD genotype and primaquine dose) was −2.9% (95% CI −5.1% to −0.60%; *p* = 0.012) in the PMQ-1 group and −2.0% (95% CI −4.1% to 0.06%; *p* = 0.056) in the PMQ-0.5 group. Corresponding values for heterozygous female participants were 1.5% (95% CI −3.0% to 6.0%; *p* = 0.503) in the PMQ-1 group and 2.4% (95% CI −2.3% to 7.0%; *p* = 0.315) in the PMQ-0.5 group.

**Table 2:** Comparison of the mean maximum individual fractional haematocrit reductions between G6PD heterozygous and wild-type females taking PMQ-1 or PMQ-0.5 not adjusted for initial parasitaemia.

Group	Mean Maximum Fractional Hct Change	95% CI	*p*-Value		
			WT PMQ-0.5 as the comparator	WT PMQ-1 as the comparator	Het PMQ-0.5 as the comparator
WT PMQ-0.5	−11.7	−13.1 to −10.3	n/a	n/a	n/a
WT PMQ-1	−11.4	−12.8 to −10.0	0.776	n/a	n/a
Het PMQ-0.5	−15.5	−19.0 to −12.1	0.045	0.135	n/a
Het PMQ-1	−23.6	−27.0 to −20.2	<0.0001	<0.0001	0.001

Hct, haematocrit; Het, heterozygote; n/a, not applicable; WT, wild type.

**Table 3:** Comparison of the mean maximum individual fractional haematocrit reductions between G6PD heterozygous and wild-type females taking PMQ-1 or PMQ-0.5 adjusted for initial parasitaemia.

Group	Mean Maximum Fractional Hct Change	95% CI	*p*-Value		
			WT PMQ-0.5 as the comparator	WT PMQ-1 as the comparator	Het PMQ-0.5 as the comparator
WT PMQ-0.5	−1.9	−3.4 to −0.48	n/a	n/a	n/a
WT PMQ-1	−1.8	−4.4 to 0.69	0.919	n/a	n/a
Het PMQ-0.5	−5.6	−9.5 to −1.6	0.058	0.137	n/a
Het PMQ-1	−13.9	−17.8 to −10.0	<0.0001	<0.0001	0.001

The median interval to the nadir of haematocrit reduction was 3 d (IQR 2 to 5) in wild-type female participants in the PMQ-1 group and 3.5 d (IQR 2 to 6) in the PMQ-0.5 group. In heterozygous female participants, the corresponding values were 5 d (IQR 4 to 6) in both groups.

### Haemolysis and Quantitative G6PD Activity

**Fig 4:**
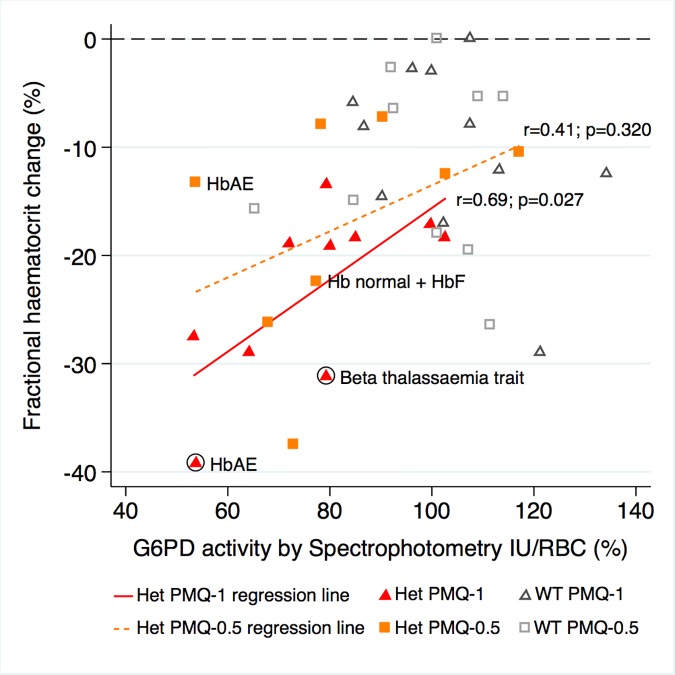
Correlation between maximum individual fractional haematocrit reduction and mean G6PD activity (IU/RBC)*. *Three G6PD heterozygous females with missing data. The difference between the regression lines for heterozygote PMQ-1 and heterozygote PMQ-0.5 was not statistically significant (*p* = 0.433). The haemoglobin type was normal unless otherwise indicated. Circled shapes represent participants who received a blood transfusion.

G6PD activity was measured in 21 heterozygous females and 21 control wild-type females at steady state, i.e., at least 6 wk after recruitment when the participants had recovered fully. After controlling for initial parasitaemia and including tests for interaction between G6PD activity and genotype (the interaction term between G6PD genotype and primaquine dose was not significant, so it was not included in the model), there was no correlation between the mean individual fractional haematocrit reduction and G6PD activity expressed as IU/gHb within any group. However, when expressed in IU/RBC, there was stronger evidence of a relationship. In the PMQ-1 group, the reduction in haematocrit for heterozygous females was 3.1% less (95% CI 0.66% to 5.44%; *p* = 0.014) for a 10% higher level of G6PD activity and 3.2% less (95% CI 0.83% to 5.54%; *p* = 0.009) in the PMQ-0.5 group (Fig 4).

### Symptom Reporting and Adverse Events

The incidence of anaemia requiring haematinic treatment within the first 42 d from enrolment, defined as an absolute haematocrit <30%, was not different between heterozygote females who received PMQ-1 (11/17 [65%]) or PMQ-0.5 (6/16 [38%]) (OR 2.1 [95% CI 0.47 to 9.3]; *p* = 0.328). When comparing G6PD heterozygous and wild-type females in the PMQ-1 group, the heterozygotes were significantly more likely to require haematinic treatment (OR 8.9 [95% CI 2.6 to 30.6]; *p* = 0.001). A similar result was found when comparing G6PD heterozygous females in the PMQ-1 group with the wild-type females in the PMQ-0.5 group (OR 5.3 [95% CI 1.68 to 16.4]; *p* = 0.004). The only participants requiring blood transfusion were two heterozygous females who received PMQ-1; recovery occurred without further complications (Table 4). Active and passive reporting of symptoms potentially associated with haemolysis (headache, backache, abdominal pain, muscle pain, dizziness, palpitations, and fatigue) during study drug administration was similar in the different groups. Abdominal pain was not different between participants in the PMQ-1 compared to the PMQ-0.5 groups (OR 1.9 [95% CI 0.71 to 5.0], *p* = 0.201).

**Table 4:** Characteristics of two G6PD heterozygous participants who required transfusion.

Case	Age (y)	Drug Group	G6PD FST Result	Day 0 Hct	Day 0 Parasitaemia per μL	First Day of Maximum Fractional Hct Reduction	Maximum Fractional Hct Reduction	G6PD Spectro (IU/Hb)*	G6PD Spectro (IU/RBC)*	Hb Type
1	13	DP PMQ-1	normal	28.0%	4800	Day 5	39.3%	62%	54%	HbAE
2	21	DP PMQ-1	normal	32.0%	32153	Day 5	31.3%	99%	79%	Beta thal trait

DP, dihydroartemisinin-piperaquine; Hb, haemoglobin; RBC, red blood cell; Spectro, spectrophotometry.

* Calculated as mean of three different measurements at steady state.

## Discussion

These results show that the standard high dose primaquine regimen given over 14 d (0.5 mg base/kg/d) is reasonably well tolerated by G6PD Mahidol heterozygous female participants, whereas significant haematocrit reductions (fractional haematocrit reduction >25%) were observed in those taking the higher daily dose (same total dose) given over 7 d (1 mg base/kg/d). Two heterozygous participants receiving the higher primaquine dose given over 7 d (1 mg base/kg/d) required transfusion. G6PD Mahidol wild-type female participants did not have an increased risk of primaquine-induced haemolysis when taking the same dose. Irrespective of primaquine regimen and despite continued dosing, haematocrits increased by day 7. The lack of subjective complaints and change in vital signs suggests that haematocrit declines were tolerated and these symptoms and signs did not identify moderate or even severe degrees of haemolysis [11].

Relapse in vivax malaria is a major cause of morbidity in tropical countries and in areas of high transmission contributes to mortality in young children from severe anaemia [12,13]. Prevention of relapse (radical cure) requires use of 8-aminoquinoline drugs, with the attendant risk of haemolysis in patients who are G6PD deficient. Since primaquine was introduced 65 y ago, it has been widely recommended and widely not given. This is because screening tests for G6PD deficiency have not been available generally, and the potential risks of giving primaquine unwittingly to a deficient patient were considered to outweigh the overall benefits [14]. Those risks depend on the degree of deficiency, determined by the G6PD genotype, and the exposure to the oxidant metabolites of primaquine, determined largely by the dose and the ability to metabolise the parent compound (reflecting in part cytochrome 2D6 activity and thus the 2D6 genotype) [15,16]. However, increased deployment of radical treatment is necessary if the enormous burden of vivax malaria is to be reduced, and this is being encouraged strongly [17]. To this end, simple point-of-care tests for G6PD deficiency have been developed to guide treatment. These tests have similar performance characteristics to the conventional NADPH FST and so will reliably identify G6PD homozygous females and hemizygous males, but, as this study illustrates clearly, tests with a detection threshold of ≳30%–40% of normal activity do not identify a large proportion of female heterozygotes. There is a significant dose-dependent haemolytic risk in some of these patients [18,19].

Although there are now more than 200 different G6PD deficiency genotypes described, usually resulting in an unstable enzyme, there is substantial phenotypic variability within genotypes, so severe haemolysis can still occur with so called “mild variants.” Amongst the more common G6PD variants globally, the African A-genotype is at the mild end of the spectrum, and the Mediterranean variant is at the severe end. The Mahidol variant studied in this trial is associated with low G6PD activities in hemizygous males, suggesting it is towards the severe end of the spectrum. Oxidant haemolysis in G6PD deficiency results in loss of the most deficient cells. In most deficiencies, these are the older and mature erythrocytes in which the unstable enzyme activity has declined such that oxidant defences can no longer be maintained. As these cells are haemolysed, they are replaced by young red cells from the bone marrow with much higher levels of enzyme activity. These young erythrocytes are therefore relatively resistant to oxidant stresses and are less likely to haemolyse [20]. As a result, in all but the most severe deficiencies, there is an initial fall in haemoglobin followed by a rise despite continued drug administration [21,22]. *P*. *vivax* malaria is also associated with haemolytic anaemia. In this study, primaquine had a negligible additional haemolytic effect over that caused by *P*. *vivax* malaria in women who were wild type for the G6PD Mahidol variant. However, heterozygous females with *P*. *vivax* malaria who had screened as “normal” (which suggests at least ≳30%–40% of their red blood cell population had normal levels of G6PD activity) experienced significant additional haemolysis as a result of taking a higher dose of primaquine over 7 d (1 mg base/kg/d). Although the potential for severe haemolysis in female G6PD heterozygotes has been well recognised [23], this has been little studied. In this study, haemolysis was dose dependent, as reported previously in hemizygous males [11]. Heterozygous females who received a higher primaquine dose of 1 mg base/kg/d haemolysed significantly more and required more treatment of their anaemia than those receiving the standard high 0.5 mg base/kg/d dose. Despite this, the haematocrit started to rise again, usually after the fifth day, as the new young red cells with higher levels of G6PD enzyme activity entered the circulation. This suggests that strategies to attenuate primaquine haemolytic toxicity by dose spacing should reduce risks in G6PD heterozygotes with the Mahidol variant but that starting radical treatment with higher daily individual doses of 8-aminoquinolines is potentially dangerous if blood transfusion is not immediately available.

Unfortunately, this relatively common, potentially high-risk group is not identified by current screening tests. Even the “gold standard” spectrophotometric assay of average erythrocyte G6PD activity (expressed as IU/gHb), which certainly cannot be performed widely in the rural tropics, showed a poor correlation with haemolytic toxicity. When spectrophotometric results were expressed as IU/RBC, a correlation was evident, which suggests that quantitative methods used to measure G6PD activity should be adjusted in anaemic populations. Handheld point-of-care quantitative testing methods and other qualitative tests of G6PD deficiency are being developed with detection thresholds of ~70% activity. The performance of these new methods and the number of truly normal (i.e., wild-type genotypes) individuals these tests may misidentify as G6PD deficient remain to be assessed.

There are a number of limitations in this analysis. Firstly, the primary trial was not designed specifically to address primaquine-induced haemolysis in G6PD heterozygous females. The number of heterozygous female participants was small, and pooling the CQ and DP groups by primaquine regimens was necessary. As with chloroquine, dihydroartemisinin-piperaquine has been shown to increase primaquine plasma concentrations [24], but it is not known whether bioactive metabolites are higher or lower than chloroquine. Because of the small number of participants analysed, conclusions could not be made on the relationship of haemoglobin type with G6PD deficiency, haemolysis, and anaemia after treatment with primaquine. There was a paucity of data in heterozygous females with enzymatic activity in the range of 40% to 60% of normal who took PMQ-0.5, and the fractional haematocrit reductions during primaquine administration in this group require further confirmation.

For the present, the practical therapeutic implications of these findings are that, for areas where G6PD deficiency genotypes of similar or greater severity to G6PD Mahidol are prevalent, daily primaquine doses greater than 0.5 mg base/kg/d should not be given to female patients irrespective of the G6PD screening result unless they can be closely monitored. More information is needed on haemolytic patterns and haemolytic risk in patients with G6PD Mahidol and other G6PD deficiency genotypes and with point-of-care quantitative testing when it becomes more widely available. Safer methods of giving primaquine are needed.

## Supporting Information

S1 STROBE Checklist (PDF)

S1 Appendix Primaquine dosing table for *P*. *vivax* (liver stage)*. (PDF)

S1 Data Dataset for main analysis. (XLS)

S2 Data Dataset for adverse event analysis. (XLS)

S1 Table Daily mean fractional haematocrit changes by G6PD genotype and treatment arm. (PDF)

S2 Table Daily mean fractional haematocrit changes by G6PD genotype and pooled by primaquine dose. (PDF)

## Abbreviations

CQ: chloroquine 25 mg base/kg divided over 3 d
DP: dihydroartemisinin 7 mg/kg and piperaquine 55 mg/kg divided over 3 d
FST: fluorescent spot test
G6PD: glucose-6-phosphate dehydrogenase
Hb: haemoglobin
IQR: interquartile range
OR: odds ratio
PMQ-1: primaquine 1 mg base/kg/d for 7 d
PMQ-0.5: primaquine 0.5 mg base/kg/d for 14 d
RBC: red blood cell
rpm: rotations per minute
SMRU: Shoklo Malaria Research Unit
