# Supplementary material for: Haemolysis in G6PD Heterozygous Females Treated with Primaquine for Plasmodium vivax Malaria: A Nested Cohort in a Trial of Radical Curative Regimens
Source: PLoS Med. 2017 Feb 7;14(2):e1002224. doi: 10.1371/journal.pmed.1002224 (PMC5295665; doi:10.1371/journal.pmed.1002224)
Supplement: S1 Appendix — (PDF) [file pmed.1002224.s002.pdf]

S1 Appendix. Primaquine dosing table for *Plasmodium vivax* (liver stage)*

1 tablet contains 15 mg

Dose: (0.5 mg/kg/day) for 14 days

A suspension is made by allowing 1 tablet to dissolve in 5ml clean water (1ml=3mg)

For more accurate dosing, use suspension in children ≤ 20 kg

For children

Weight (Kg)	ml(cc)
5	0.8 ml
6	1.0 ml
7	1.2 ml
8	1.3 ml
9	1.5 ml
10	1.7 ml
11	1.8 ml
12	2 ml
13	2.2 ml
14	2.3 ml
15 - 17	2.5 ml
18 - 20	3 ml

For adults and older children

Weight (Kg)	tab
10 – 14	0.25
15 – 20	0.5
21 - 26	0.75
27 - 34	1
35 - 40	1.25
41 - 48	1.5
49 - 56	1.75
57 - 65	2
66 - 80	2.5
81 - 100	3

Use tablet cutter to cut tablets

Give food before dose to prevent abdominal pain and nausea

*This chart was taken from the SMRU Malaria guidelines, 18th edition. At lower weights, suspension was recommended for more accurate dosing. Doses were doubled for patients randomised to the primaquine 1 mg base/kg/day x 7 days (PMQ-1) groups. The primaquine dosing chart was developed specifically for this area and the dose may not be applicable to other areas.
