# Supplementary material for: Haemolysis in G6PD Heterozygous Females Treated with Primaquine for Plasmodium vivax Malaria: A Nested Cohort in a Trial of Radical Curative Regimens
Source: PLoS Med. 2017 Feb 7;14(2):e1002224. doi: 10.1371/journal.pmed.1002224 (PMC5295665; doi:10.1371/journal.pmed.1002224)
Supplement: S1 Table — (PDF) [file pmed.1002224.s005.pdf]

S1_Table. Daily mean fractional haematocrit changes by G6PD genotype and treatment arm

Fractional hct change compared to day 0	Wild type females (198)							
	CQ+PMQ-1 (47)		DP+PMQ-1 (49)		CQ+PMQ-0.5 (48)		DP+PMQ-0.5 (54)	
	Mean	SD (95% CI)	Mean	SD (95% CI)	Mean	SD (95% CI)	Mean	SD (95% CI)
Day 1	-3.3	6.3 (-5.2,-1.5)	-4.7	6.5 (-6.6,-2.9)	-3.0	9.8 (-5.8,-0.1)	-3.2	7.3 (-5.2,-1.2)
Day 2	-4.3	6.8 (-6.3,-2.2)[1]	-6.5	6.2 (-8.3,-4.7)	-5.4	7.0 (-7.5,-3.4)	-5.5	7.2 (-7.4,-3.5)
Day 3	-5.2	7.6 (-7.4,-2.9)	-6.5	6.5 (-8.3,-4.6)	-6.1	9.6 (-8.9,-3.3)	-4.8	9.3 (-7.4,-2.3)
Day 4	-4.8	7.6 (-7.0,-2.5)	-6.2	7.9 (-8.5,-3.9)	-5.0	10.3 (-8.0,-2.0)[1]	-6.0	8.7 (-8.4,-3.6)
Day 5	-3.4	7.5 (-5.6,-1.3)	-6.2	7.5 (-8.4,-4.0)[1]	-3.4	10.7 (-6.6,-0.3)[2]	-5.4	9.1 (-7.8,-2.9)
Day 6	-2.5	7.8 (-4.8,-0.1)[1]	-6.2	6.9 (-8.1,-4.2)[1]	-4.3	10.1 (-7.3,-1.2)[3]	-3.9	10.2 (-6.7,-1.1)
Day 7	-1.0	8.6 (-3.6,1.6)[2]	-5.2	7.8 (-7.4,-2.9)[1]	-2.0	10.3 (-5.0,1.1)[2]	-4.5	10.5 (-7.4,-1.6)[1]
Day 14	-0.3	8.0 (-2.7,2.2)[4]	-4.5	10.2 (-7.6,-1.4)[5]	-2.7	10.4 (-5.8,0.4)[2]	-2.8	10.7 (-5.8,0.2)[2]

Fractional hct change compared to day 0	Heterozygous females (33)							
	CQ+PMQ-1 (6)		DP+PMQ-1 (11)		CP+PMQ-0.5 (11)		DP+PMQ-0.5 (5)	
	Mean	SD (95% CI)	Mean	SD (95% CI)	Mean	SD (95% CI)	Mean	SD (95% CI)
Day 1	-4.3	2.6 (-7.0,-1.5)	-3.9	5.3 (-7.5,-0.3)	-0.8	6.3 (-5.0,3.4)	-0.3	11.5 (-14.5,14.0)
Day 2	-9.1	5.5 (-14.9,-3.4)	-8.3	7.5 (-13.3,-3.3)	-4.7	5.5 (-8.6,-0.8)[1]	-3.5	8.9 (-14.6,7.6)
Day 3	-7.5	6.3 (-14.0,-0.9)	-15.4	11.3 (-22.9,-7.8)	-6.1	7.2 (-11.0,-1.2)	-11.6	4.6 (-17.3,-5.9)
Day 4	-16.4	7.9 (-24.6,-8.1)	-14.0	16.0 (-25.4,-2.5)[1]	-8.0	7.2 (-12.8,-3.2)	-14.5	4.4 (-19.9,-9.0)
Day 5	-15.8	8.8 (-25.0,-6.6)	-23.2	11.0 (-31.0,-15.3)[1]	-11.9	11.1 (-19.4,-4.4)	-11.0	5.2 (-17.5,-4.5)
Day 6	-14.4	8.4 (-23.3,-5.6)	-21.9	9.1 (-28.5,-15.4)[1]	-11.6	8.7 (-17.4,-5.7)	-16.5	7.4 (-25.7,-7.3)
Day 7	-12.8	8.6 (-21.8,-3.8)	-18.8	8.4 (-24.9,-12.8)[1]	-10.8	7.5 (-15.8,-5.8)	-13.2	7.3 (-22.3,-4.1)
Day 14	-3.6	9.1 (-13.1,5.9)	-6.2	6.1 (-17.0,4.7)[4]	-5.5	8.5 (-11.2,0.2)	-4.0	12.9 (-20.1,12.0)

Results expressed as mean (standard deviation)
Superscript is the number of subjects with missing data
Primaquine 1 mg base/kg/day x 7 days (PMQ-1), Primaquine 0.5 mg base/kg/day x 14 days (PMQ-0.5)
