# Supplementary material for: Haemolysis in G6PD Heterozygous Females Treated with Primaquine for Plasmodium vivax Malaria: A Nested Cohort in a Trial of Radical Curative Regimens
Source: PLoS Med. 2017 Feb 7;14(2):e1002224. doi: 10.1371/journal.pmed.1002224 (PMC5295665; doi:10.1371/journal.pmed.1002224)
Supplement: S2 Table — (PDF) [file pmed.1002224.s006.pdf]

S2_Table. Daily mean fractional haematocrit changes by G6PD genotype and pooled by primaquine dose

Fractional hct change compared to day 0	Wild type females (198)				Heterozygous females (33)			
	PMQ-1 (96)		PMQ-0.5 (102)		PMQ-1 (17)		PMQ-0.5 (16)	
	Mean	SD (95% CI)	Mean	SD (95% CI)	Mean	SD (95% CI)	Mean	SD (95% CI)
Day 1	-4.0	6.4 (-5.3,-2.7)	-3.1	8.5 (-4.8,-1.4)	-4.0	4.5 (-6.3,-1.7)	-0.6	7.8 (-4.8,3.5)
Day 2	-5.4	6.6 (-6.7,-4.1)[1]	-5.5	7.1 (-6.8,-4.1)	-8.6	6.7 (-12.0,-5.1)	-4.3	6.5 (-7.9,-0.7)[1]
Day 3	-5.8	7.0 (-7.2,-4.4)	-5.4	9.4 (-7.3,-3.6)	-12.6	10.3 (-17.9,-7.3)	-7.8	6.9 (-11.5,-4.1)
Day 4	-5.5	7.7 (-7.1,-3.9)	-5.5	9.4 (-7.4,-3.7)[1]	-14.9	13.3 (-21.9,-7.8)[1]	-10.0	7.0 (-13.8,-6.3)
Day 5	-4.8	7.6 (-6.4,-3.3)[1]	-4.5	9.8 (-6.4,-2.5)[2]	-20.4	10.6 (-26.0,-14.8)[1]	-11.6	9.5 (-16.7,-6.6)
Day 6	-4.3	7.5 (-5.9,-2.8)[2]	-4.1	10.1 (-6.1,-2.0)[3]	-19.1	9.4 (-24.1,-14.1)[1]	-13.1	8.4 (-17.6,-8.6)
Day 7	-3.1	8.4 (-4.9,-1.4)[3]	-3.3	10.4 (-5.4,-1.2)[3]	-16.6	8.7 (-21.2,-11.9)[1]	-11.6	7.3 (-15.4,-7.7)
Day 14	-2.4	9.3 (-4.4,-0.4)[9]	-2.7	10.5 (-4.9,-0.6)[4]	-5.0	10.2 (-11.2,1.2)[4]	-5.1	9.6 (-10.2,0.1)

Results expressed as mean (standard deviation)
Superscript is the number of subjects with missing data
Primaquine 1 mg base/kg/day x 7 days (PMQ-1), Primaquine 0.5 mg base/kg/day x 14 days (PMQ-0.5)
